# Supplementary material for: Cytokines TNFα, IFNγ and IL-2 Are Responsible for Signal Transmission from the Innate Immunity Protein Tag7 (PGLYRP1) to Cytotoxic Effector Lymphocytes
Source: Cells. 2020 Dec 4;9(12):2602. doi: 10.3390/cells9122602 (PMC7761954; doi:10.3390/cells9122602)
Supplement: Supplementary file 1 [file cells-09-02602-s001.pdf]

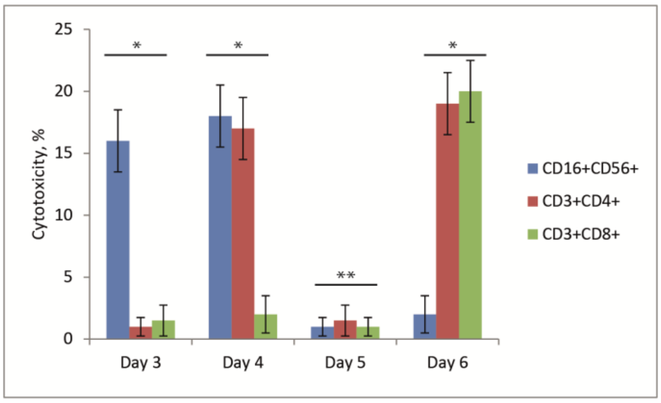

Supplementary Fig. S1. Changes in cytotoxic activity of different lymphocyte subpopulations induced in PBMC incubated with Tag7 (10^{-9}M) for different periods of time. Lymphocyte subpopulations were isolated by magnetic cell separation and tested for cytotoxicity against K562 cells. Data are presented as the mean ± SD of 3 independent experiments. Differences from the control in all cases are significant at * $p < 0.03$, ** $p < 0.05$ (Student t test).

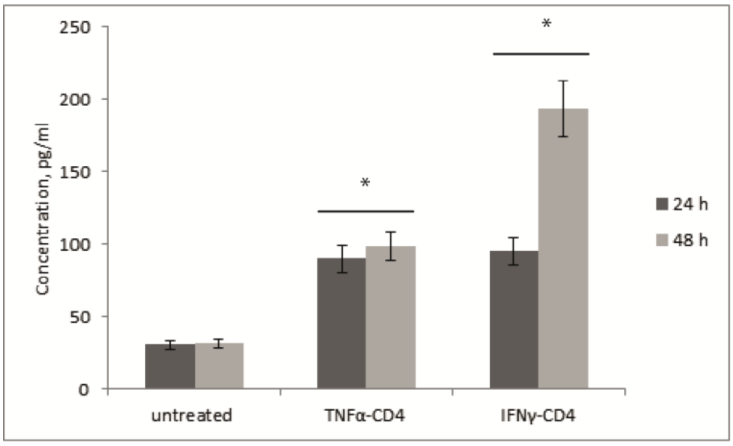

Supplementary Fig. S2. Secretion of IL-2 by $CD3^{+}CD4^{+}$ - lymphocytes incubated with TNFα or IFNγ for 24 and 48 hours. $CD3^{+}CD4^{+}$ - lymphocytes were isolated by magnetic cell separation from PBMC on 0 day and treated with TNFα or IFNγ. Conditioned medium from untreated lymphocytes was used as a control. The medium was sampled every 24 h to determine IL-2 level by ELISA. Data are presented as the mean ± SD of 3 independent experiments. Differences from the control in all cases are significant at * $p < 0.05$ (2-way ANOVA).

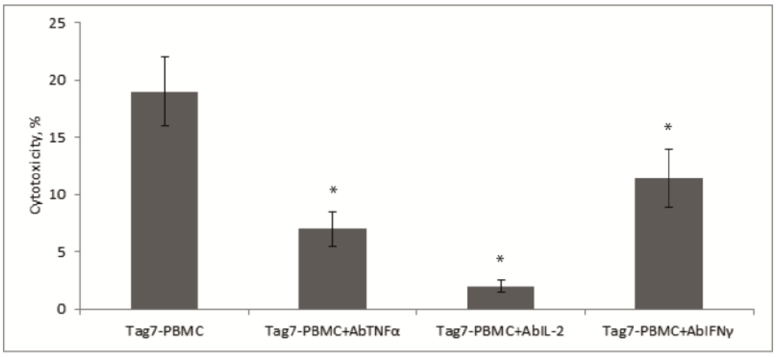

Supplementary Fig. S3. Cytotoxicity Tag7-PBMC in presense specific antibodies to TNFα, IFNγ, IL-2. Each antibody (1:1000) was added to PBMC before 1h to Tag7 (10^{-9}M). After 6 days of incubation PBMC were tested for cytotoxicity against K562 cells. Data are presented as the mean ± SD of 3 independent experiments. Differences from the control in all cases are significant at * $p < 0.05$ (Student t test).
